# Race and ethnicity: Risk factors for fungal infections?

**DOI:** 10.1371/journal.ppat.1011025

**Published:** 2023-01-05

**Authors:** Jeffrey D. Jenks, Chioma Inyang Aneke, Mohanad M. Al-Obaidi, Matthias Egger, Lorena Garcia, Tommi Gaines, Martin Hoenigl, George R. Thompson

**Affiliations:** 1 Durham County Department of Public Health, Durham, North Carolina, United States of America; 2 Division of Infectious Diseases, Department of Medicine, Duke University, Durham, North Carolina, United States of America; 3 Department of Laboratory Medicine, National Institutes of Health, Bethesda, Maryland, United States of America; 4 Department of Veterinary Pathology and Microbiology, University of Nigeria, Nsukka, Nigeria; 5 Division of Infectious Diseases, Department of Medicine, University of Arizona, Tucson, Arizona, United States of America; 6 Division of Infectious Diseases, Medical University of Graz, Graz, Austria; 7 Department of Public Health Sciences, UC Davis School of Medicine, Davis, California, United States of America; 8 Division of Infectious Diseases and Global Public Health, Department of Medicine, School of Medicine, University of California, San Diego, California, United States of America; 9 University of California Davis Center for Valley Fever, Sacramento, California, United States of America; 10 Department of Internal Medicine, Division of Infectious Diseases, University of California Davis Medical Center, Sacramento, California, United States of America; 11 Department of Medical Microbiology and Immunology, University of California Davis, Davis, California, United States of America; Rutgers University, UNITED STATES

## Abstract

Racial and ethnic identities, largely understood as social rather than biologic constructs, may impact risk for acquiring infectious diseases, including fungal infections. Risk factors may include genetic and immunologic differences such as aberrations in host immune response, host polymorphisms, and epigenomic factors stemming from environmental exposures and underlying social determinants of health. In addition, certain racial and ethnic groups may be predisposed to diseases that increase risk for fungal infections, as well as disparities in healthcare access and health insurance. In this review, we analyzed racial and ethnic identities as risk factors for acquiring fungal infections, as well as race and ethnicity as they relate to risk for severe disease from fungal infections. Risk factors for invasive mold infections such as aspergillosis largely appear related to environmental differences and underlying social determinants of health, although immunologic aberrations and genetic polymorphisms may contribute in some circumstances. Although black and African American individuals appear to be at high risk for superficial and invasive *Candida* infections and cryptococcosis, the reasons for this are unclear and may be related to underling social determinants of health, disparities in access to healthcare, and other socioeconomic disparities. Risk factors for all the endemic fungi are likely largely related to underlying social determinants of health, socioeconomic, and health disparities, although immunologic mechanisms likely play a role as well, particularly in disseminated coccidioidomycosis.

## Introduction

Differences in biological sex are known factors that increase the risk of acquiring a number of infectious diseases, including invasive fungal infections, which largely have a male predilection. In one recent review of this topic, all invasive fungal infections except candidiasis were shown to be overrepresented in biological males, ranging from invasive aspergillosis (IA) (51% males overall, although most studies in the literature report a larger male predominance) to cryptococcosis (74% males) [1]. Factors that explain the male predominance of invasive fungal infections may include differences in steroid hormone homeostasis, sex-specific immune response, behavioral factors such as occupational exposure, medical comorbidities such as human immunodeficiency virus (HIV), and gender disparities in health care, among others [1].

Much less is known about the relationship between race and ethnicity and risk of acquiring invasive fungal infections. Admittedly, this is a complicated, yet essential, question to address. Categorizing individuals into racial groups, as has historically been reported in prior publications, is complicated as race is now understood to largely be a social rather than a biologic construct [2–5]. While historically racial categories have existed as a means to categorize common hereditary traits, such as skin color, there is more genetic variability within racial groups than between them. In a seminal paper published in 1972, Richard Lewontin showed that 85.4% of genetic diversity within humans occurs within populations in a racial group, 8.3% of variation between populations within racial groups, and only 6.3% of genetic variation between racial groups [6]. In addition, grouping individuals into broad racial and ethnic categories is equally fraught. For example, an individual born in Cuba with West African ancestry may identify their ethnicity as Hispanic or Latino, but feel their lived experience is very different from other Cuban nationals who identify as white. Thus, drawing conclusions between race and ethnicity (social constructs) and human genetics (a biologic construct) is complex and multi-faceted, prompting professional organizations such as the American Medical Association to provide guidance on the use of race, ethnicity, and genomics in medical and scientific literature [5,7].

With these limitations in mind, here we review the literature on racial and ethnic differences and risk of invasive fungal infection caused by molds, yeast, and selected endemic mycoses in the United States (US). The review was performed within the context that race and ethnicity categorizations are incredibly complicated entities and that multiple non-biologic factors may influence risk of invasive fungal infections, both between and within racial and ethnic groups.

## Results

### Human genomic ancestry and predisposition to fungal infections

Fungi are opportunistic pathogens and range from ubiquitous organisms (e.g., *Aspergillus* spp.) with frequent inhalational exposure, to commensals (e.g., *Candida* spp.) requiring a breach in the normal protective barriers of the skin or gastrointestinal system, to the endemic fungi (e.g., *Histoplasma* and *Coccidioides* spp.) present within specific geographic regions. The host immune response must simultaneously kill invading fungal organisms, while minimizing the surrounding inflammatory reaction and maintaining immune homeostasis [8].

The constitutive mechanisms of immunity are displayed at locations with frequent interaction with fungal pathogens including the mucosa of the respiratory epithelium and the gastrointestinal tract [9]. Host defensins, collectins, and the complement system provide nonspecific defense and recognition of fungi. These pathways are highly conserved; however, medication-induced complement defects have been associated with IA [10]. Host-cell expression of pattern recognition receptors, including Toll-like receptors (TLRs) and C-type lectin receptors (CLRs) sense pathogen-associated molecular patterns (PAMPs) are present in fungi [9]. Numerous single-nucleotide polymorphisms (SNPs) within human TLRs (TLR1 [11], TLR4 [12–15], TLR6 [11], among others) and CLRs (Dectin-1—in particular the Y238X SNP [16–18]; DC-SIGN, mannose receptor and mannose-binding lectin [19–22]) have been identified as risk factors for fungal disease. DC-SIGN and pentraxin 3 have been identified as key macrophage receptors assisting in the recognition and phagocytosis of fungal species with deleterious polymorphisms identified [23–25].

Following fungal recognition and phagocytosis, intracellular killing occurs by the generation of NADPH-dependent reactive oxidant species (ROS). Defects within this pathway (e.g., chronic granulomatous disease) exhibit heightened susceptibility to both bacterial and some fungal pathogens. Additional signaling pathways initiating antifungal immunity have also been identified, but not yet fully characterized, including the calcium-calcineurin-NFAT pathway.

Neutrophils are well known for their role in providing protection against invading fungal pathogens and their recruitment is dependent upon chemokine release [26], and polymorphisms resulting in CXCL10 expression changes have been found associated with aspergillosis [27]. Subsequent SYK-CARD9 signaling induces the inflammasome and results in the activation of proinflammatory cytokines. Defects in the CARD9 pathway have been found predisposing to chronic mucocutaneous candidiasis [28] and disseminated aspergillosis [29], while polymorphisms in a number of cytokines have also been found to predispose to fungal disease: aspergillosis (IL-1 [30], IL-10 [31,32], IL-15 [32], and IL-23); candidiasis (IL-4) [33,34]; paracoccidioidomycosis (IL-4) [35]; and blastomycosis (IL-6) [36]. Polymorphisms within the tumor necrosis factor-α (TNF-α) gene [32] and its receptors [37,38] have also been associated with susceptibility to aspergillosis. Polymorphisms within the interferon-γ (INF-γ) gene [39] and its receptor have also been associated with fungal disease risk, and auto-antibodies to IFN-γ have similarly seen an increased rate of fungal infections and may be more frequent in some patient groups [40].

Host neutrophils also release antimicrobial peptides (e.g., defensins) and proteases, and attempt to sequester iron availability in response to fungal invasion [8,41]. Plasminogen also appears to be a key regulator in susceptibility to aspergillosis [42]. Polymorphisms, including changes in copy number, may confer changes in susceptibility and are the source of ongoing investigation.

Extensive investigation of T cells over the last 3 decades has further enhanced our understanding in their role in providing protective immunity [43]. Chronic noninvasive forms of aspergillosis such as asthmatic exacerbations, allergic bronchopulmonary aspergillosis, and chronic pulmonary aspergillosis are also defined by aberrant T-cell responses. A dominant Th2 response is observed in allergic type diseases, while a proinflammatory phenotype has been described in those with chronic forms of pulmonary aspergillosis and improvements in our understanding of this complex and highly coordinated immune response may lead to the recognition of immunogenetic factors responsible.

An association of the immunogenetic factors responsible for protection or susceptibility to invasive fungal diseases with genomic ancestry has been demonstrated for only a few diseases and seems to be most clear with disseminated coccidioidomycosis. The aforementioned SNPs in IL-6 associated with blastomycosis susceptibility appear to be overrepresented in the Hmong population conveying increased risk [36]. The Y238X Dectin-1 polymorphism associated with increased fungal risk is overrepresented in some populations [16] and may be responsible for the increased risk for fungal diseases observed in certain demographic groups. Human leukocyte antigens (HLA) class II antigens (HLA-A9 and HLA-B9 antigens) and ABO blood type B have been associated with more severe coccidioidomycosis infection, although it is unclear if there is a causal association or if this association is merely due to an increased proportion of these phenotypes in Filipino and black or African American individuals [44,45]. In addition, in another study the HLA class II-DRB1*1301 allele was a marker for disseminated coccidioidomycosis, independent of race or ethnicity [46].

Recent work analyzing the association between environmental exposure, psychosocial stressors, and nutrition has found these important influences in the patient genome and epigenome over multiple generations [47]. These social determinants of health may lead to alterations in patient DNA methylation, chromatin remodeling, histone modification, and regulatory RNA changes and subsequently alter the patient immune response [48]. The epigenetic changes may alter cell type-specific and temporal gene expression ultimately resulting alterations of individual patient risk for fungal infections [49]. There have been few studies evaluating the epigenetic changes that may be at play in susceptibility to fungal disease; however, this remains a promising potential area of inquiry and is a key area of investigation that may help explain differing risk for invasive fungal disease between patient groups

#### Social determinates of health and fungal infections

In the US, certain racial and ethnic groups may endure a greater burden of fungal disease due to underlying comorbidities including HIV, diabetes, and hematologic malignancies. For example, African Americans and Latinos accounted for nearly 71% of all new HIV diagnoses in 2019, but only represent 30% of the overall US population [50]. Significant racial and ethnic disparities in the incidence and survival of hematologic malignancies have also been documented [51,52]. Furthermore, racial and ethnic differences in diabetes and corresponding complications have been thoroughly discussed in the literature and are clear risk factors for IFIs [53–55].

Race and ethnicity are socially constructed terms without a biological basis in the scientific literature [5,56]. Rather, racial inequities observed in fungal disease rates are likely driven by the circumstances in which individuals are born, live, work, and age [57]. Intersectionality is a framework that helps us understand how socialized categories like race and ethnicity interact with other social factors such as gender, socioeconomic status, occupation, and employment, combine, overlap, and interact to create health inequalities in the US [58,59]. For example, Coronavirus Disease 2019 (COVID-19) mortality data initially pointed to biologic sex differences as men died at higher rates than women, but these differences were moderated by race, ethnicity, geographic location within the US, and time [60]. As further data emerged, it pointed to a complex intersection of social factors, similar to other coronavirus pandemics, that included race, ethnicity, occupation, socioeconomic status, and the social determinants of health [61–65]. Pulmonary aspergillosis, an invasive fungal disease, is well known to cause a superinfection in critically ill patients with COVID-19 infection, particularly those with underlying comorbidities such as hypertension, COPD, and HIV [66–69]. These comorbidities, as previously noted, are also associated with health care access and utilization, poverty, race, and ethnicity, similar to other social determinants of health that interacted to shape COVID-19 health disparities in the US.

Notably, communities of color are disproportionately affected by poorer social, economic, and environmental conditions that can contribute to elevated vulnerabilities from fungal infections. Healthcare accessibility has previously been discussed by our team as a plausible explanation of gender disparities in invasive fungal infections [1]. Regarding racial and ethnic disparities, US census data demonstrates that African Americans, Latinos, American Indians, and Alaska Natives have higher rates of being uninsured relative to white individuals [70]. Uninsured adults are more likely than those with insurance to postpone healthcare or forgo it altogether, leading to greater inequities in the delivery of healthcare. Moreover, despite the implementation of US policies to reduce healthcare disparities, recent research covering a 20-year timespan demonstrated that African Americans and Latinos continue to experience more barriers to healthcare services compared with white individuals [71].

### Differences in race/ethnicity in investigated pathogens

#### Molds

***Aspergillosis*.** *Aspergillus* spp. can cause a spectrum of disease in humans, from non-invasive diseases such as allergic bronchopulmonary aspergillosis that are associated with exacerbation of different underlying lung diseases, to life-threatening infections such as invasive pulmonary aspergillosis, including in those with COVID-19 infection [8,67,68,72]. Climate factors play a role in the airborne spread of the spores that can vary based on regions and seasons [73] and more importantly, can result in selection pressure for antifungal resistant ones, especially in regions that have exposure to azoles pesticides [74]. Therefore, to understand any association between *Aspergillus* spp.-related disease and race as a risk factor, one should understand the underlying risk factors of those diseases and their racial propensities.

IA has a high risk of morbidity and mortality, and can present as an invasive disease of the lung and the sinuses, and in rare cases present as a disseminated disease, often related to immunodeficiencies, most of which are acquired [8]. Indeed, the risk of developing IA is highest among patients with hematological malignancies, recipients of hematopoietic stem cells (HSCT) and solid organ transplants (SOT) recipients. Racial minorities suffer from hurdles accessing healthcare, including accessing solid and hematopoietic transplants [75,76]; therefore, there may be a hypothetical increase in the incidence of IA among the white population who receive these procedures more often. For example, a retrospective study of a transplant registry found that black patients were less likely to receive heart transplant than white patients (aHR 0.87, 95% CI 0.84 to 0.90) and had a higher risk of post-transplant death (aHR 1.14, 95% CI 1.04 to 1.24) [75]. Similarly, black or African Americans experience a longer time on the renal transplant waiting list [77] and are less likely to complete a kidney transplant compared to white Americans [78,79]. However, most studies evaluated the epidemiology of IA in immunosuppressed patients showed either a minor increase in the incidence of IA among the black or African American population (44.6 in black or African American versus 42.9 in white populations per 1 million persons) [80] or no association with propensity score matching [81], which signals the lack of evidence of race association and IA in high risk immunosuppressed patients.

Taking underlying anatomical pathologies as risk for aspergillosis, there may be an association of such diseases with certain hereditary and pathological diseases, one of those diseases is cystic fibrosis (CF), which is highly linked to the white population [82]. This can be seen in the finding that white CF patients have higher risk for persistent *Aspergillus* spp. colonization (OR 1.74, 95% CI 1.23 to 2.48, *p* = 0.002) than black or African American CF patients [83]. Such findings could be related to worse CF disease in white CF patients than in black or African American CF patients. In contrast, allergic fungal sinusitis, which can be caused by *Aspergillus* spp., has a higher incidence in the southern regions of the US, especially among black or African American compared to white populations living in these regions [84], which signals environmental and possibly socioeconomic factors related to the development of this disease.

Climatic factors were suggested to influence IA incidence rates among HSCT patients. This hypothesis was tested in a study of IA from large transplant centers in Seattle, Washington and Houston, Texas, which showed increased incidence rates of IA during summer months among HSCT in Seattle than in non-summer months. These findings were likely due to the higher burden of *Aspergillus* spp. spores in the air during the drier warm season following the rainy seasons, but such finding was not visible in the Houston center’s cohort [73]. While findings that certain regions in the US, especially the western states, have a higher incidences of IA [80], there was no statistical difference in the incidence of IA in different parts of the US after implementing propensity matching [81]. However, those studies did not evaluate seasonal differences. The association of seasonal effect and IA incidence should be further studied, especially as climate change may accentuate such regional and seasonal differences related to IA incidences and could place certain racial groups at a higher risk for IA versus others.

Lastly, azole-resistant *Aspergillus* spp. is an emerging pathogen that is linked to the use of agricultural azoles (tebuconazole and propiconazole) has been reported in different parts of the world. The incidence of such azole-resistant *Aspergillus* spp. in the US remains low, but it was reported from crop debris in the southeast regions of the US, with isolates carrying TR_46_/Y121F/T289A mutations that are significantly linked to azole resistance [74]. Such environmental presence of azole-resistant *Aspergillus* spp. in places where low socioeconomic populations live, including racial minorities, places those individuals at risk of acquiring invasive disease that is difficult to treat.

***Mucormycosis and other rare molds*.** While mucormycosis occurs worldwide [85], the vast majority of cases are reported from India and neighboring regions, where there is limited racial disparity [86,87]. In India, a steep increase of mucormycosis cases was observed during the COVID-19 pandemic [88,89], driven by specific immunological mechanisms that predispose COVID-19 patients to mucormycosis [87], as well as overuse of systemic corticosteroids and an increase in the population with undiagnosed or uncontrolled diabetes [88]. Outbreaks of mucormycosis outside of India are often associated with natural disasters [90] such as hurricanes [91,92], tsunamis [91], or floodings [93], primarily affecting socially disadvantaged populations living in affected areas. For example, after 1 tornado in Missouri, US in 2011, 13/13 cases of necrotizing cutaneous mucormycosis occurred in white individuals [92]. Other outbreaks of mucormycosis have been associated with contaminated hospital products such as linen or a wooden spatula [94,95]. Race was not a factor reported in the vast majority of these outbreak descriptions. Among transplant recipients in the US who developed mucormycosis, white race was predominant (90.5%) [96], while other large epidemiological studies from the US failed to report on racial distribution [97].

Prevalence of other rare mold infections such as fusariosis, lomentosporiosis, scdeosporiosis, and phaeohyphomycosis vary between geographical regions. For example, lomentosporiosis occurs primarily in Australia, Southwestern Europe, and Southwestern US [98]. Unfortunately, the majority of the larger studies, whether reporting cases from around the world [99–102] or from specific geographical regions [103–105], fails to report race and ethnicity of its participants. Larger outbreaks in the US were often associated with contaminated hospital products, such as an *Exserohilum rostratum* outbreak in patients receiving contaminated methylprednisolone injections [106]. Among 65 cases of invasive fusariosis in the US and Canada, 78.5% were white, 9.2% Asian, 6.2% black, and 4.6% Hispanic ethnicity [107]. Among 99 cases of phaeohyphomycosis (62 from the US, 7 from Australia, and 7 from Peru), 68% identified as white, 14% Hispanic/Latino, 8% Asian, and 7% black; the proportion of phaeohyphomycosis cases who were white further increased in the subgroup of disseminated disease (77%), while Hispanic/Latino cases represented 28% of those with local-superficial disease [108]. Among transplant recipients in the US, the vast majority developing fusariosis (94.4%) or scedosporiosis (91.3%) were white [96].

#### Yeast

***Candida infections*.** *Candida* species can cause invasive infections in humans, including bloodstream infection or deep-seated infection, or a non-invasive disease mainly involving mucocutaneous infections [109]. Several *Candida* species are identified as a cause of infections in humans and animals, with the most commonly identified organisms including *C*. *albicans*, *C*. *glabrata*, *C*. *krusei*, *C*. *tropicalis*, *C*. *parapsilosis*, and *C*. *kefyr*. The epidemiology of different infections varies from hospital-related infections—in the cases of invasive disease—to non-invasive infections that can affect non-hospitalized patients, such as with mucocutaneous candidiasis [109,110].

Several risk factors are related to invasive candidiasis, such as immunosuppression and surgical procedures [109]. In a study of candidemia in 4 US states from 2012 to 2016, black or African American individuals had higher rates of invasive candidiasis compared to those who didn’t identify as black or African American (rate 2.3 (95% CI: 2.1 to 2.6) [111]. While such findings can be confounded by regional racial distribution, this risk was adjusted to the geographical location, which included sites from Georgia and Maryland that had a black or African American population of around 40%, and sites from Oregon and Tennessee, with a black or African American population less than 10% [111]. A similar finding from another study evaluating the burden of candidemia in the US, included data from 9 US states. It found that although overall rates of candidemia across these sites were 7.0 cases per 100,000 persons, the highest rates were in black or African American individuals (12.3 cases per 100,000 persons), with about a quarter of all cases in black or African American individuals [112]. Other similar findings were reported in an observational study that found that racial minority groups had a higher risk for *Candida* endophthalmitis than white patients (OR 1.65, 95% CI 1.07 to 2.55) [113]. Possible factors for the high incidence rate of candidemia in black or African American individuals include factors related to socioeconomic status, underlying medical conditions, and healthcare access [111]. Besides rates of invasive candidiasis associated with certain racial minorities, selection of certain *Candida* spp. may be associated with race groups, as reported among transplant patients with invasive candidiasis, black or African American individuals were noted to have a higher incidence compared to white individuals of *C*. *glabrata*; however, further studies are needed to confirm such observation and evaluate the mechanism of such risk [114].

In cases of non-invasive candidiasis, black or African American women were more likely (11.5%) to have colonization from *Candida* spp. compared to Hispanic (9.8%) or white women (8.5%) [115]. Black or African American women also had a 7-fold higher risk of having vulvovaginal candidiasis (VVC) than other racial groups among university students [116], in 1 study. A similar finding of a higher incidence of self-reported physician diagnosed VVC among black or African American women than among white women in a telephone survey study [117]. Inversely, white individuals with HIV had higher rates of oral candidiasis compared to black or African American men [118].

Overall, such race and candidiasis relationships are presented in an epidemiological manner that does not explain the pathology behind such association, and the likelihood of confounding factors related to healthcare access and socioeconomic status is very high.

***Cryptococcosis*.** *Cryptococcus neoformans* and *Cryptococcus gattii* are 2 species complexes that are the etiological agents of nearly all human and animal cryptococcosis [119]. Separation of strains using molecular markers into various serotypes, varieties, and groups reveal that *C*. *gattii* is an etiological agent of cryptococcosis in immunocompromised individuals status post organ transplantation, rheumatic immune diseases, diabetes mellitus, and malignancies as well as in healthy individuals [120]. *C*. *gattii* affects HIV-uninfected persons in tropical and subtropical regions while *C*. *neoformans* primarily affects persons with HIV infection worldwide [121]. The epidemiology of cryptococcosis changed significantly with the rise and fall of the AIDS pandemic and emergence of various pathogenic *Cryptococcus* spp. since the 1980s.

Human hosts are infected following the inhalation of spores that subsequently invade pulmonary alveoli, causing pulmonary diseases, or that disseminate through the bloodstream, often leading to fatal meningitis [122–124]. Cryptococcal meningitis (CM) associated with HIV infection is one of the leading opportunistic infections [125], and mortality from cryptococcosis ranges from 8% to 50% [126]. There has been an increasing interest in *C*. *gattii* infections over the past 2 decades due to the emergence of *C*. *gattii* in the Pacific Northwest region of the US. In July 2010, 60 human cases were reported to the Centers for Disease Control and Prevention (CDC) from 4 states (California, Idaho, Oregon, and Washington) in the Pacific Northwest [127].

In an analysis of deaths from cryptococcosis among individuals living with HIV infection in the US from 1999 to 2016, with respect to race, there were 199 deaths from cryptococcosis among women with HIV infection, 38 deaths among non-Hispanic white individuals, and 161 among black or African American individuals. Non-Hispanic white individuals had significantly lower mortality rates than black or African American individuals, with a mortality rate in black or African American men of 0.19 (95% CI 0.17 to 0.21) and 0.06 in black or African American women (95% CI 0.05 to 0.06). The mortality rate in white men and women was <0.001 [128]. In a single center, retrospective study of individuals with cryptococcosis admitted from October 2005 to October 2017 [129], of 114 patients admitted to the University of Kentucky HealthCare Medical Center, males made up 74.6% (85/114) of patients and 91.2% (104/114) were white. Cryptococcosis in Hispanic persons and black or African American persons was more common in the HIV-infected group compared to the transplant and non-HIV/non-transplant (NHNT) groups (*p* < 0.0001). Among HIV-infected persons in a US survey, the incidence of cryptococcosis in 1993 was significantly higher among black or African American persons (31/1,000) than among white persons (23/1,000; relative risk [RR] = 1.3, 95% CI, 1.1 to 1.6) [130]. In another study [131] investigating the prevalence of undiagnosed cryptococcal infection among HIV-infected person in the US from 1986 to 2012, stored sera from 1,872 participants in the Multicenter AIDS Cohort Study and the Women’s Interagency HIV Study were screened. Of those specimens, the overall presence of cryptococcal antigen (CrAg) positivity was 2.9%, with no significant differences observed in the proportion of CrAg-positive specimens by race and ethnicity, except in persons of “other” ethnicity (i.e., not white (2.5%), black (2.5%), or Hispanic (1.7%)) had a prevalence of 6.4% (CI = 3.9% to 10.3%).

#### Endemic mycoses in the United States

***Coccidioidomycosis*.** *Coccidioides* spp. (*C*. *imitis* and *C*. *posadasii*) is a dimorphic fungus endemic to the southwestern US. It grows in the environment in the mycelial form, and the yeast form infects the animal host after inhalation and leads to coccidioidomycosis [132,133]. Most people who get infected do not develop symptoms, and only a small minority, about 1%, may develop disseminated disease [134]. However, it is thought that around 25% of all community-acquired pneumonia in endemic regions is secondary to coccidioidomycosis [135].

Several epidemiological studies reported higher incidence rates of coccidioidomycosis among racial and ethnic minorities in the US endemic regions [136–138]. This was shown in a recent CDC study using data of reportable endemic mycoses from 26 states that found that American Indian/Alaska Native (AI/AN) cases and Hispanic cases had higher incidence rates of coccidioidomycosis (17.4 and 11.2 per 100,000, respectively) compared to non-Hispanic white cases (4.1 per 100,000). However, data on race and ethnicity were available in less than half (39%) of the reported cases [138]. In addition, geography and regional exposure to dust might have influenced coccidioidomycosis rates in different racial and ethnic groups. Such a hypothesis was explored in coccidioidomycosis surveillance in California (1973 to 2011) that showed the increase in the incidence of coccidioidomycosis followed different environmental exposures, including occupational exposures such as construction and agriculture, regardless of the population’s racial and ethnic groups [139]. Such geographically related trends can vary based on regional analysis, as the Hispanic population had a much higher incidence than white populations in areas such as San Joaquin Valley [140], which may be associated with the type of occupation and outdoor recreational activities among different racial and ethnic groups.

Moreover, high levels of particulate matter with diameters less than 10 micrometers (PM_10_) in certain parts of Arizona have been associated with an increased risk of coccidioidomycosis, and such regional high PM_10_ exposure is more likely to affect black or African American and Hispanic populations [141]. Another factor to account for is the knowledge gaps among Hispanic farm workers about coccidioidomycosis [142] that can make recognizing the disease and providing early treatment challenging. Moreover, while black or African American inmates in California had higher rates of symptomatic coccidioidomycosis than white inmates [143,144], a study using skin tests to screen for coccidioidomycosis showed no association between coccidioidomycosis and race [142]. However, in the latter study the number of inmates who agreed to the skin test was smaller among minority racial and ethnic groups compared to white inmates. Thus, the association between racial and ethnic groups and the acquisition of coccidioidomycosis are likely related to socioeconomic and health disparities factors, which are likely to influence the incidence rates in these populations.

These factors should be taken into account as we learn that climate change will likely influence the spread of coccidioidomycosis endemicity to different geographic regions. Such environmental spread may, in turn, expose certain racial or ethnic groups more than others to this infection [133,145]. Moreover, environmental injustices, in which poor communities and communities of color are disproportionately exposed to environmental harms yet environmental protections are limited [146], could further contribute to higher rates of coccidioidomycosis among racial and ethnic groups.

Older age, diabetes mellitus, and immunosuppression are some of the risk factors for developing severe and disseminated coccidioidomycosis [147]. It was observed that certain racial and ethnic groups were more likely to develop severe and disseminated coccidioidomycosis, such as Asians (especially Filipinos), and black or African Americans were reported to be at increased risk for coccidioidomycosis complications [148–150]. AI/AN was reported to have an increased risk of coccidioidomycosis complications, but among this population, case fatality rates were shown to have trended down between 1959 and 1980, with unchanged coccidioidomycosis incidence rate over the same period, which may be explained by the change in the social, economic, environmental and the availability of new therapies for coccidioidomycosis during that time period [151].

Black or African American and Filipino individuals were reported to be at a higher risk compared with white individuals of hospitalization from pulmonary coccidioidomycosis [152], with several reports alluding that black or African American individuals have the highest associated mortality, dissemination, and hospitalizations, with odds of disseminated disease as high as 5 to 10 times the rates seen among the white individuals [137,152–155] and this increased risk persisted after controlling for income [156]. Among the immunocompromised patients, the risk of symptomatic coccidioidomycosis rates shown to be higher among black or African American individuals with HIV infection compared to white individuals [157], but this relationship was not seen in the renal transplant recipients with coccidioidomycosis [158]. This could be secondary to different populations’ underlying immunocompromising conditions and other factors related to socioeconomic status and health disparity of those 2 populations that likely confounded the relationship. Also, black or African American individuals with coccidioidomycosis were found to have lower rates compared to white individuals of erythema nodosum, which is an immunological response to the infection that is thought to be protective against coccidioidomycosis [159,160], which may hint at the higher rates of disseminated and severe disease in this population.

In conclusion, it is not well established if genetic predispositions are the main driver of increased risk of coccidioidomycosis and certain racial and ethnic groups, although there is a clear increased risk of severe coccidioidomycosis infection in certain racial or ethnic groups, such as Filipinos and black or African Americans. Factors such as socioeconomic status, high inoculum exposure, and healthcare access with delay in the diagnosis may contribute to this increase in disease severity, along with predisposing genetic factors, as previously noted.

***Histoplasmosis*.** *Histoplasma capsulatum* is a dimorphic fungus with at least 4 cryptic species. *Histoplasma capsulatum sensu stricto* is endemic to Panama and the northern portion of South America [161] while *Histoplasma suramericanum* is distributed widely across South America. *Histoplasma mississippiense* is distributed in the Mississippi River Valley and *Histoplasma ohiense* in the Ohio River Valley, both in the US [162]. Locally acquired infections outside these areas in the US have been reported, showing that the geographic range of histoplasmosis in the US is wider than is often appreciated [163].

Following the inhalation of spores of the soil-dwelling dimorphic *Histoplasma* spp., only a minority of individuals develop symptomatic disease [164]. In 2019, the CDC received 1,124 case reports of histoplasmosis from 12 states where it is a reportable disease. The overall incidence of histoplasmosis in these states was 1.8 cases per 100,000 population, including Illinois (292 cases (26%), rate: 2.3 cases per 100,000 persons), Michigan (225 cases (20%), rate 2.3 cases per 100,000 persons), and Minnesota (214 cases (19%), rate 3.8 cases per 100,000 persons). These 3 states accounted for a combined 65% of these cases [138].

Occupational exposures are frequently implicated in histoplasmosis outbreaks. Early in the HIV pandemic, reports of *Histoplasma* spp. outbreaks [164–166] demonstrated an increased risk of death associated with advanced AIDS (CD4 counts <75 mm3), immunocompromised states (such as solid organ transplantation), chronic renal disease, and prolonged use of corticosteroids or tumor necrosis factor (TNF) antagonists [164,166,167].

From 2011 to 2014, a total of 3,409 histoplasmosis cases were reported from 12 states where histoplasmosis is reportable. Of the 1,729 patients in 8 states that contributed race data, 1,079 (62%) were white, 446 (26%) were of unknown race, and 166 (10%) were black. Of the 1,620 patients in these 8 states for whom ethnicity data were available, 1,072 (66%) were non-Hispanic or Latino, 503 (31%) were of unknown ethnicity, and 45 (3%) were Hispanic or Latino. Mortality data was available for 1,142 patients, of which 76 (7%) died [168]. In the 2019 survey, of 1,124 cases, there was data on race and ethnicity in 859 (76%) of cases. Of these cases, 656 (76%) of cases occurred in white persons, with incidence highest in white persons (1.3 per 100,000 population), AI/AN (1.2), and Hispanic persons (1.2). Of those which hospital data was available for (460 cases), 249 (54%) persons were hospitalized, and 20 (5%) persons died. In this survey, histoplasmosis incidence was similar across racial and ethnic categories (range: 0.9 to 1.3) [138].

Although anyone can acquire histoplasmosis in areas where *Histoplasma* spp. is present in the environment, persons living with advanced HIV are at a particularly high risk for developing histoplasmosis. Clinical signs and symptoms of this disease are often nonspecific, making it difficult to establish a diagnosis unless the index of suspicion is high. Complications of disseminated histoplasmosis, including adrenal insufficiency, endovascular infection, meningitis, and hemophagocytic lymph histiocytosis, are uncommon but challenging to manage [169].

***Blastomycosis*.** Blastomycosis is an uncommon but underdiagnosed and potentially life-threatening infection caused by the dimorphic fungi *Blastomyces dermatitidis*, which includes at least 1 cryptic subspecies, *B*. *gilchristii* [170]. These organisms live in warm, moist soil with plentiful organic matter. It is endemic throughout much of the midwestern US [171,172], particularly along the Great Lakes and the Mississippi, Ohio, and Saint Lawrence River valleys [173,174]. The region of geographic risk for blastomycosis is incompletely understood for multiple reasons, including the difficulty pinpointing the time of exposure in some patients who have a long clinical latency period, the absence of a skin test or other well-known marker of prior exposure, and lack of instances in which *Blastomyces* spp. have been recovered from the environment. A survey of 240 case reports of blastomycosis from 5 states (Arkansas, Louisiana, Michigan, Minnesota, and Wisconsin) in 2019 showed that the overall blastomycosis incidence in these states was 0.8 cases per 100,000 population, with Minnesota (rate 1.4) and Wisconsin (rate 1.7) accounting for 179 (75%) of the total cases [138]. Endemicity is most pronounced in the hyperendemic regions of north central Wisconsin where the disease incidence can exceed 100 cases per 100,000 inhabitants [175]. The geographic range appears to be shifting, with new regions at risk including New York state [172].

Blastomycosis mainly affects immunocompetent persons, although immunocompromised persons are more likely to develop more severe forms of the disease [176]. In a survey reporting 4,441 blastomycosis cases in 5 US states from 1987 to 2018, 2,778 (64%) occurred in white persons, 740 (17%) in persons of unknown race, 406 (9%) in black or African American persons, and 193 (5%) in Asian, Native Hawaiian, or other Pacific Islander persons. The majority of persons, 2,828 (71%), did not identify as Hispanic or Latino and ethnicity was unknown for 1,015 (26%) persons [173]. A study in central and northern Wisconsin found that while 90% of blastomycosis cases in non-Hispanic white persons were caused by *B*. *dermatitidis*, *B*. *gilchristii* frequently caused infection in Hispanic white, AI/AN, and Asian persons.

Furthermore, while non-Hispanic white persons were frequently older and had more underlying medical conditions compared to Hispanic white and Asian persons, the odds of hospitalization were 2 to 3 times higher for Hispanic white, AI/AN, and Asian persons [177]. In another study of persons admitted to the University of Mississippi Medical Center and treated for blastomycosis from 1980 to 2000, there was a clear predominance of black or African American men admitted to the hospital, followed by black or African American women. Among the 123 hospitalized persons, 100 (81%) were black or African American, 21 (17%) white, and 2 (2%) Native American. White females were least likely to be hospitalized with blastomycosis in this study [178]. The increased risk for hospitalization among racial and ethnic minorities may signify blastomycosis-related health disparities [178–180]. Differences in genetic composition have been postulated to underlie ethnic disparities in incidence rates of this endemic blastomycosis [36], including one study showing case clustering among persons of Hmong ethnicity [171].

## Conclusion

**Fig 1:**
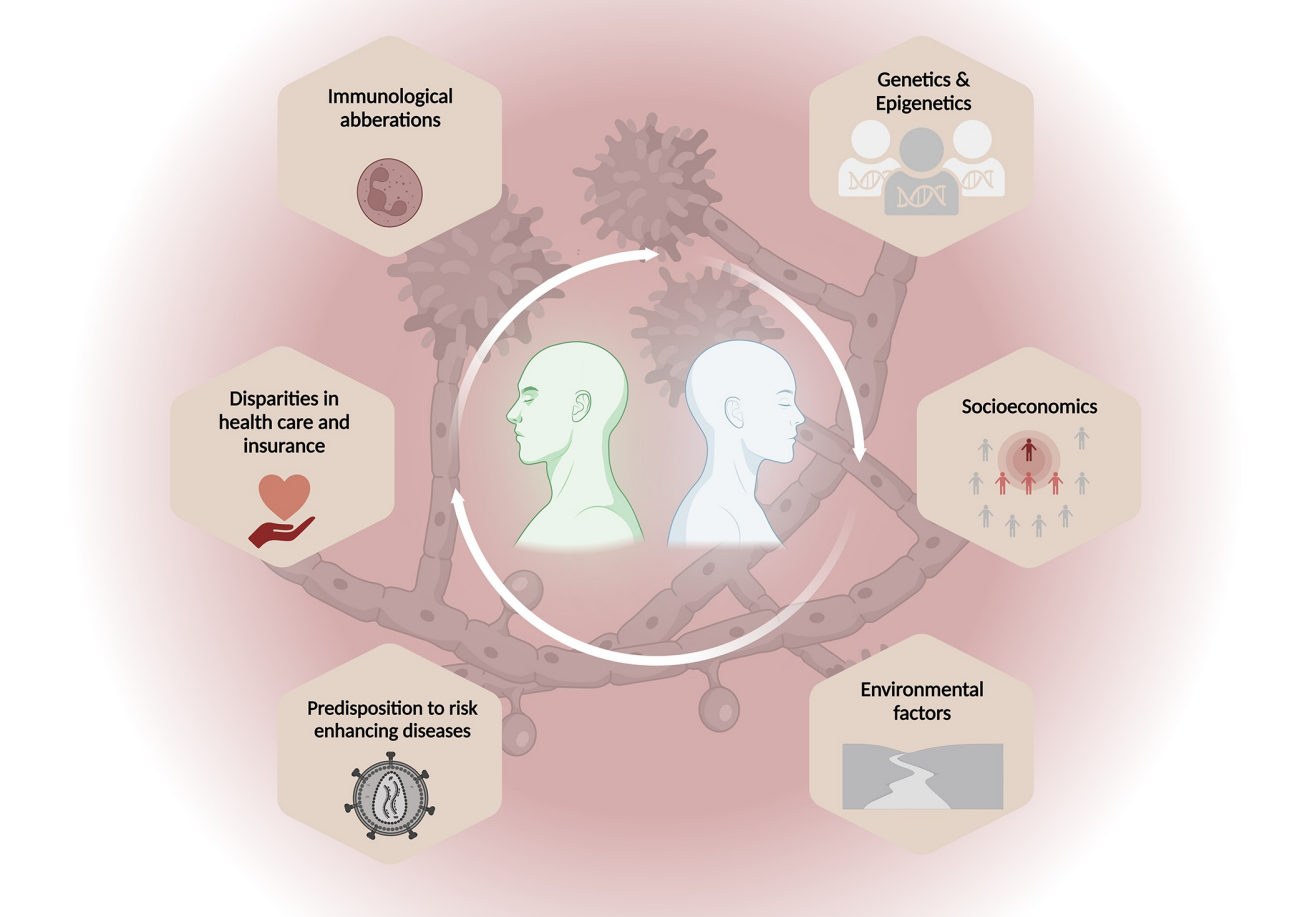
Factors that may explain differences in racial and ethnic distribution in fungal diseases. The figure was created with BioRender.com.

Although now largely understood as a more social than biologic construct, racial and ethnic identity may impact risk for acquiring infectious diseases, including fungal infections. Risk factors for fungal infections may include genetic and immunologic risk factors such as aberrations in host immune response, host polymorphisms, and epigenomic factors that may stem from underlying social determinants of health. In addition, social determinants of health and underlying socioeconomic factors may increase risk for fungal infections as certain racial and ethnic groups may be predisposed to diseases that increase risk for fungal infections, as well as disparities in healthcare access and health insurance (**Fig 1**). In this review, we analyzed race and ethnicity as risk factors for acquiring fungal infections as well as race and ethnicity as they relate to risk for severe disease from fungal infections.

Risk factors for aspergillosis and other invasive mold infections largely appear related to environmental differences and underlying social determinants of health, although immunologic aberrations and genetic polymorphisms may play a role in some circumstances, such as defects in the CARD9 pathway and polymorphisms such as with IL-1, Il-10, IL-15, IL-23, TNF-α, and INF-γ. Conversely, although black or African American individuals appear to be at a higher risk compared to white individuals for superficial and invasive *Candida* infections as well as cryptococcosis, the reasons for this are unclear from an immunologic/genetic standpoint, and may therefore be rather related to underling social determinants of health, access to healthcare, and other socioeconomic disparities.

Native American/American Indian and Hispanic/Latino populations are at a higher risk compared to white populations of coccidioidomycosis infection, although it is likely that this is at least partially related to occupational and environmental exposure, as individuals in these populations are more likely to engage in outdoor occupation that puts them at risk for coccidioidomycosis and also reside in areas where coccidioidomycosis is endemic. Certain populations, such as Filipinos and black or African American populations, are at increased risk compared to white populations for severe or disseminated coccidioidomycosis. The exact reason for this risk is unclear, but several immunologic mechanisms may contribute to the occurrence of more severe disease, such as the presence of HLA-A9 and HLA-B9 antigens, ABO blood type B, HLA class II-DRB1*1301 allele.

Data on race and ethnicity for histoplasmosis and blastomycosis are further complicated based on their geographic distribution and the fact that neither of these diseases is nationally reportable. As with the other fungi, risk factors for severe disease may be related to underlying social determinants of health, socioeconomic, and health disparities. Underlying immunologic mechanisms may contribute as well, such as polymorphisms in IL-6 that have been observed to be overrepresented in the Hmong population and been postulated risk factor for blastomycosis infection in this population [36].

Attributing disease risk to genetic factors across racial and ethnic groups is fraught considering the large genetic variability within these populations, although as previously mentioned, there may be specific genetic and immunologic factors that predispose individuals within populations to infection or severe disease. For most fungal diseases, other factors that may affect certain racial or ethnic groups more than others, such as environmental exposures and differences in underlying social determinants of health may explain differences observed in epidemiology and disease severity. Further investigation is necessary to elucidate epigenetic changes due to psychosocial stressors, environmental exposures, or other underlying social determinants of health as a risk factor for fungal infections.
